# Triple-negative breast cancer, pregnancy, and HLA alloreactivity: a cause for concern?

**DOI:** 10.3389/fonc.2026.1806495

**Published:** 2026-04-13

**Authors:** Giovanna Crestani Morassutti, Maurizio Zanetti

**Affiliations:** 1 Freelance Writer, Friuli, San Vito al Tagliamento, Italy; 2 The Laboratory of Immunology, Department of Medicine and Moores Cancer Center, University of California San Diego, La Jolla, CA, United States

**Keywords:** alloreactivity, genetic, HLA, surrogacy, TNBC

## Abstract

Triple-negative breast cancer (TNBC) remains the most aggressive form of breast cancer in women worldwide, with an incidence that varies considerably depending on genetic background and ethnicity. Since parity is a known risk for TNBC, here we propose that in HLA alloreactivity resulting from immunization against paternal antigens expressed by the fetus, 50% fetus–mother HLA dissimilarity in natural pregnancy and 100% fetus–mother HLA dissimilarity in surrogate motherhood, may be a risk factor in women with predisposing genetic background and ethnicity due to immune suppression and immunologic tolerance vis-à-vis a developing breast cancer with TNBC characteristics.

## Introduction

Breast cancer is a leading cause of mortality and morbidity among females worldwide, with an estimated 2.3 million new cases and 685,000 deaths, representing 12.5% of the total number of new diagnosed cases of cancer worldwide in 2020 (1). Triple-negative breast cancer (TNBC) is a subtype of invasive breast cancer characterized by the lack of estrogen receptor (ER), progesterone receptor (PR), and HER2 expression, and typically exhibits a basal-like pattern gene expression profile (2). In the US and in Europe, TNBC accounts for 10%-15% of all diagnosed breast cancers, but higher incidence is found among young women and multiparous women (3, 4). TNBC lacks approved targeted therapies, has high risk of recurrence, and has lower survival rates than ER+ breast cancer.

TNBC incidence varies among countries and populations of diverse ethnicity, suggesting genetic heterogeneity as a risk factor. Notably, approximately 20% of TNBC patients carry germline *BRCA1/2* mutations (5), which have a population frequency of about 1:400, with the exception of populations bearing high-frequency founder mutations, such as Ashkenazi Jewish (6). In the United States, TNBC incidence also varies among White women (10%-15%) and African American and Latin American women (20%-30%). TNBC among women in West Africa nations such as Ghana and Nigeria has an even greater incidence (40%-56%) (7). In India, the incidence of TNBC is also high (30%-45%) with a peak (73%) in premenopausal women under 35 years of age (8, 9).

Reproductive history has been linked with risk of breast cancers since 1970, but TNBC and ER+ breast cancers are affected differently. Nulliparity is associated with a 39% lower risk of TNBC compared with a 35% higher risk of ER+ disease in postmenopausal women (10). Conversely, parity increases the risk for TNBC (11) with a positive correlation between number of births and TNBC incidence (10), suggesting an intriguing association between TNBC risk and parity.

While genetic background and ethnicity are established risk factors for TNBC, the role of immune dysregulation as a distinct risk factor remains relatively poorly understood. It has been known for over half a century that multiparous women develop antibodies due to partial fetus–mother antigenic, mismatch alloantibodies. These antibodies are commonly directed against red blood cell antigens and histocompatibility antigens of the human leukocyte antigen (HLA) system (12). HLA alloantibodies are induced during the first trimester of pregnancy, and their incidence increases with parity (3, 13, 14). Alloreactive T cells are also activated by paternal HLA antigens expressed by the semi-allogeneic fetus. However, to a successful pregnancy, maternal T cells exposed to paternal alloantigens expressed by the fetus are kept in check by tolerance and immune suppression at the maternal–fetal interface. Mechanisms of immune control include regulatory T cells and placental expression of the non-classical HLA molecule HLA-G, an immune suppressive molecule with limited polymorphism that acts as a major immune checkpoint (15). In addition, the success’ of natural pregnancy is favored by serum mixed lymphocyte reaction blocking factor (MLR-BF), which was identified as a short-lived IgG3 alloantibody (16). MLR-BF inhibits T-cell proliferation in response to paternal alloantigens and hence immune tolerance during pregnancy (17), and it can be induced therapeutically by maternal immunization with paternal leukocytes (18). This suggests that maternal immune recognition of paternal antigens may serve as a fail-safe mechanism against spontaneous abortion, counteracting fetus–mother HLA dissimilarity. Recent findings also show that reciprocal HLA-DR allogenicity between mother and child positively affects pregnancy outcome parameters (19), implying some transient local benefits from HLA dissimilarity. Notwithstanding the apparent paradox, HLA alloantibodies and alloreactive T cells are determinants of success in natural pregnancy but may be a hitherto unappreciated risk factor in TNBC, which is the prevalent gestational breast cancer type (20).

## Hypothesis

Based on the foregoing, we propose that HLA-alloimmune history (i.e., exposure to non-self HLA molecules) may represent a potential risk or modifying factor in the development or immune biology of TNBC. Mechanistically, HLA alloantibodies are expanded by successive pregnancies on re-encounter of the same allogeneic HLA antigens expressed by the fetus, much like canonical recall (memory) immune response by B lymphocytes following vaccination or infection (21). Even though the titer of HLA alloantibodies diminishes with time in the absence of further fetal alloantigen stimulation, alloreactive memory B cells persist (22). Likewise, alloreactive T cells are also primed during natural pregnancy and persist postpartum in the majority of pregnant women for several years (23). However, they lose their vigor due to exhaustion when restimulated by fetal alloantigens (24, 25). In mice, pregnancy imprints regulatory T cells to sustain anergy to fetal antigens (26), and in humans male fetal cells may persist in blood and tissues for years “fetal maternal chimerism” (27, 28), providing for memory alloreactive T cells maintenance through antigen re-encounter. A self-maintaining alloreactivity-driven path to immune suppression is destined to complement other forms of local immune dysregulation, e.g., immune dysregulation induced by aneuploid cancer cells (29). Aneuploidy is a chromosomal abnormality prominent and ubiquitous in TNBC (30).

Whereas parity increases the risk of TNBC (11), clinical outcome of parous vs. nulliparous women with TNBC at 50% HLA dissimilarity in a natural pregnancy is influenced by lactation and breastfeeding, both providing a significant overall survival advantage after TNBC diagnosis perhaps due to an increase of intra-tumor T cells (31). Protection by lactation was shown in early studies where lactation induced in virgin mice is *per se* sufficient to inhibit mammary tumors (32). In humans, absence of or short (<6 months) breastfeeding period is an established reproductive risk factor for TNBC, with an estimated population attributable fraction of about 12% in White women and 15% in Black women in the United States (33). Although a relationship between lactation and HLA alloimmunity has not been tested directly, a longer breastfeeding period was reported to be associated with a decreased alloimmunization against non-inherited maternal antigens (34). How breastfeeding antagonizes an HLA alloreactivity-driven state of immunologic tolerance and mitigates TNBC risk is unclear. Some known factors include (a) reduced levels of estrogen and progesterone, (b) mammary tissue remodeling during pregnancy and reduced persistence of undifferentiated mammary stem cells, and (c) milk components acting as a countermeasure to local immune tolerance and tumor cell differentiation. Milk components may include anti-idiotypic antibodies against HLA-alloreactive antibodies (35), and miRNAs with either immune regulatory properties or the ability to prevent oncogenic programs in mammary cells of women at risk of breast cancer. One potential miRNA is miR-335, a potent disruptor of the oncogenic program in human TNBC cells *in vitro* and *in vivo* (36–38).

**Figure 1:**
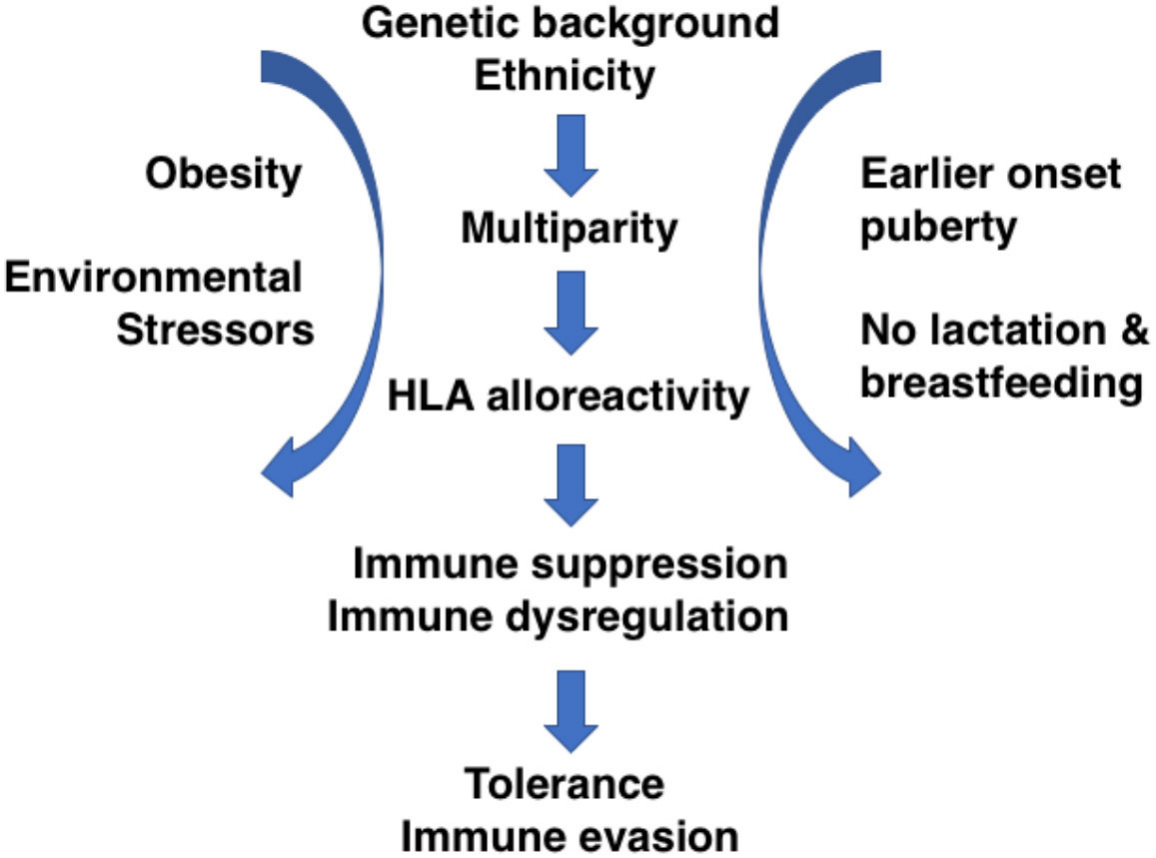
Diagram of hypothetical series of events stemming from HLA alloreactivity based on fetus–mother HLA mismatch during pregnancy leading to immune suppression, immunologic tolerance, and immune evasion in TNBC. The immunologic axis is shown in the context of other recognized factors predisposing to TBNC or contributing to its progression (see also text).

Known non-genetic risk factors for TNBC that “weave together” with HLA alloreactivity and lack of lactation include obesity, chronic mammary inflammation, activation of inducible COX-2, upregulation of checkpoint inhibitors, mutations in DNA repair genes, and chromosomal instability and aneuploidy (30, 39, 40). Interestingly, PD-L1 upregulation and activation of inducible COX-2 that are responsible for immune dysregulation are both associated with activation of the IRE1 branch of the unfolded protein response (41, 42), suggesting a vulnerability that could be exploited therapeutically. Thus, HLA alloimmunity establishes a state of immune suppression that together with other risk factors converges on a complex immune dysregulation causing immune evasion. Arguably, the immunological effects stemming from HLA alloimmunity represent only one component of a more complex scenario (**Figure 1**).

## Implications

**Figure 2:**
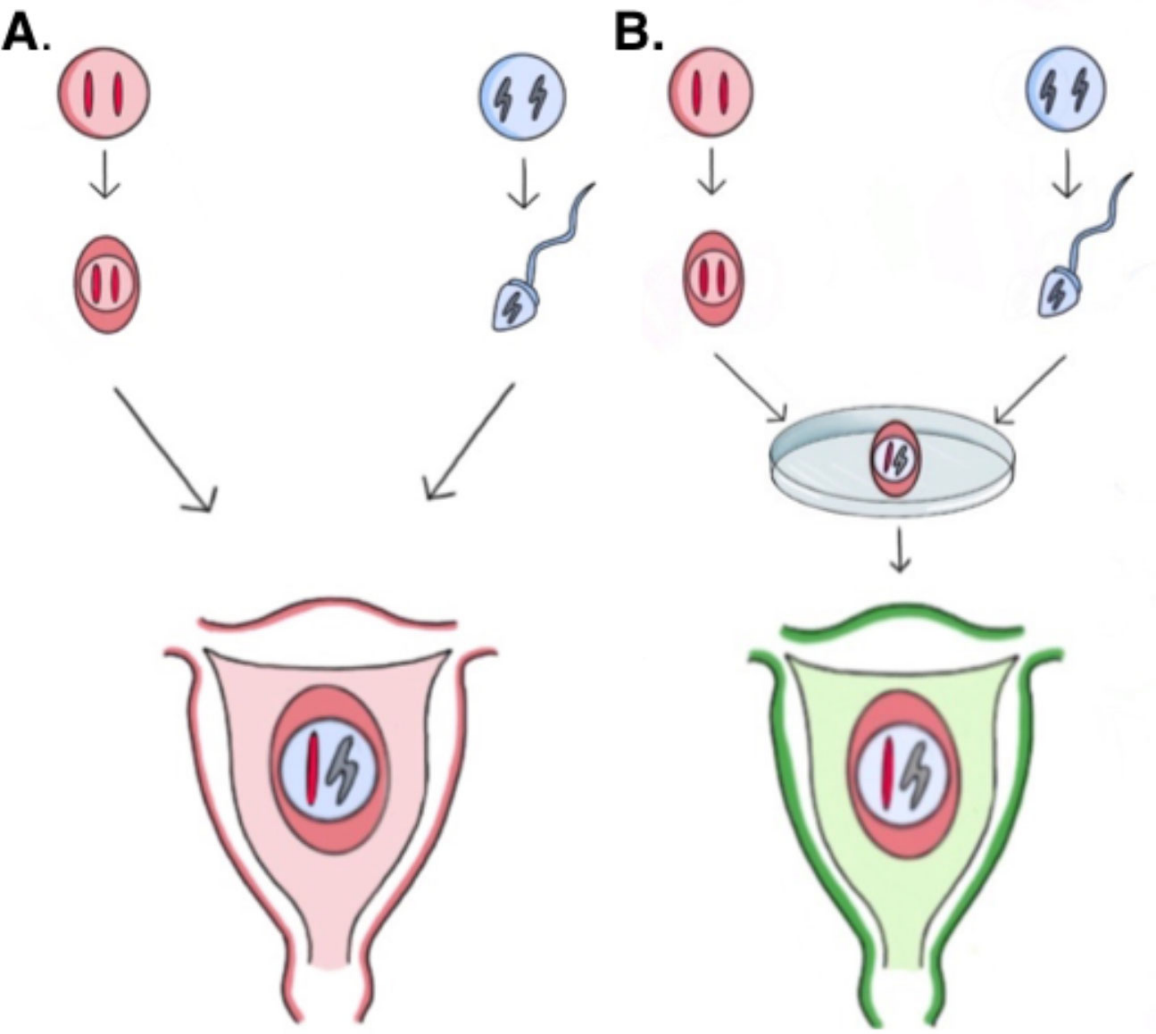
Schematic representation histocompatibility HLA antigen fetus–mother relationship in **(A)** 50% HLA mismatch and **(B)** 100% HLA mismatch. Colors identify the histocompatibility origin of the zygote in relationship to the host mother.

Paradoxically, an increased risk of triple-negative breast cancer among women with multiple recent births coexists with a sustained global decline in fertility rates, potentially reshaping the future population burden of this breast cancer subtype. The World Health Organization reports that approximately 17.5% of the global adult population—equating to one in six people—experiences infertility (affecting both males and females), with prevalence showing only modest regional variation. Since the first IVF baby in 1978, assisted reproductive technologies (ART) have evolved from infertility treatments to a reproductive norm beyond infertile couples. Third-party contributors—egg donors, sperm donors, embryo donors, and gestational surrogates—have become routine. In gestational surrogacy, surrogates are routinely implanted with embryos created *in vitro* from unrelated third-party gametes, resulting in 100% HLA mismatch between surrogate and fetus. Compared with a semi-allogeneic fetus–mother 50% HLA mismatch of a natural pregnancy, the long-term consequences of implanting a zygote (fertilized egg) created with gametes from two genetically-unrelated donors implanted into a 100% HLA mismatched host mother can be predicted easily (**Figure 2**).

Thus, a stronger alloimmunization from 100% fetus–mother HLA mismatch may promote higher chronic inflammation in placental structures, which may affect immunological tolerance at the fetus–mother interface. One cannot help but wonder about the long-term immunological consequences (strong immune suppression and tolerance). Indeed, HLA alloimmunization generates a robust T-cell tolerance barrier since survival among living donor liver transplant allografts is poorer among mothers who received the organ from their offspring as compared to unrelated living donors (43). Defective immune surveillance in TNBC also aligns with the modest clinical benefits of immune checkpoint blockade (ICB) monotherapy in these patients (44). From this, one could reasonably predict a higher risk of TNBC in multiparous women implanted with a 100% allogeneic HLA dissimilar zygote. In this context, immune suppression would need to be heightened to dampen alloreactive T cells in the setting of a 100% HLA-mismatched fetus–mother pair and to enable a successful term pregnancy.

In a fluid global surrogacy industry, the demand for surrogacy by intended parents is primarily from more economically developed countries purchasing surrogacy services from women in less developed countries (45) such as India, West African nations, and some Latin America nations, where less regulated systems permit easy and cheaper access to surrogacy. Oddly, these countries have higher TNBC incidence rates. One may then wonder whether, on these considerations, surrogacy could represent a greater risk factor for TNBC, a risk that may be further amplified by the absence of breastfeeding, a protective factor consistently associated with reduced TNBC risk in epidemiologic studies (46).

## Conclusions

We highlighted a complex interplay between alloreactivity and parity as a new risk factor for TNBC and showed how a program designed to prevent fetal rejection during pregnancy may morph into a hitherto unappreciated risk factor for TNBC in women with predisposing genetic background and ethnicity. Because TNBC lacks approved targeted therapies, a validation of the hypothesis presented herein may provide new therapeutic solutions and suggest preventive measures in women with predisposing genetic background and ethnicity for TNBC.

## Data Availability

The original contributions presented in the study are included in the article/supplementary material. Further inquiries can be directed to the corresponding author.
